# Virulence Signatures and Biofilm-Forming Capacity of ESBL-Producing *Escherichia coli* in Wastewater and Agricultural Environments

**DOI:** 10.3390/microorganisms14102239

**Published:** 2026-10-03

**Authors:** Monica S. Sellars, Courtney W. Reggans, Ajah A. Alford, Alyssa J. Harris, Jordan C. Deutschlander, Mabel Kamweli Aworh

**Affiliations:** 1 Department of Biological and Forensic Sciences, Fayetteville State University, Fayetteville, NC 28301, USA; msellars2@broncos.uncfsu.edu (M.S.S.); creggans@broncos.uncfsu.edu (C.W.R.); aalford10@broncos.uncfsu.edu (A.A.A.); aharris54@broncos.uncfsu.edu (A.J.H.); jdeutschlander@uncfsu.edu (J.C.D.); 2 Department of Population Health and Pathobiology, College of Veterinary Medicine, North Carolina State University, Raleigh, NC 27607, USA

**Keywords:** Biofilm formation, ESBL-producing *E. coli*, virulence genes, wastewater treatment plants, environmental dissemination, antimicrobial resistance

## Abstract

The persistence of extended-spectrum β-lactamase (ESBL)-producing *E. coli* in livestock and municipal wastewater systems represent a key environmental pathway for antimicrobial resistance (AMR) and virulence determinants with direct public-health implications. This study investigated the distribution of virulence genes and biofilm-forming capacity of ESBL-producing *E. coli* from two municipal WWTPs, a poultry farm, and a cattle farm. A total of 452 environmental samples were collected between May 2025 and May 2026. ESBL-producing *E. coli* were identified using culture-based and biochemical methods. Virulence genes were characterized by polymerase chain reaction (PCR) and whole-genome sequencing (WGS). Biofilm formation was assessed using the crystal violet microtiter plate assay. Of the 452 samples, 120 (26.5%) yielded ESBL-producing *E. coli*. PCR analysis showed that poultry farm isolates exhibited the highest prevalence of virulence genes, predominantly *traT* (64/66), *ompA* (61/66), *fimH* (54/66), and *chuA* (39/66), followed by isolates from WWTP-A, the cattle farm, and WWTP-B. Significant differences among sampling sources were observed for *traT* (*p* = 0.0398) and *stx1* (*p* = 0.0224). WGS analysis identified significant source-dependent associations for several virulence genes, with the strongest associations observed for *esp* (FDR = 3.47 × 10^−6^) and *fimH* (FDR = 2.36 × 10^−5^). Most isolates were non-biofilm producers (52/120, 43.3%). Among biofilm-forming isolates, weak biofilm production predominated across all sampling locations, with the highest prevalence observed in poultry farm isolates (21/120, 17.5%), followed by isolates from the cattle farm and WWTP-A (10/120, 8.3% each) and WWTP-B (7/120, 5.8%). These findings identify poultry farms and wastewater environments as important reservoirs of virulence-associated ESBL-producing *E. coli* and support integrated One Health surveillance to limit the environmental spread of AMR.

## 1. Introduction

*Escherichia coli* is a rod-shaped, Gram-negative bacterium belonging to the family Enterobacteriaceae [1]. It is a common inhabitant of the lower intestinal tract of warm-blooded animals and is frequently released into the environment through feces and wastewater effluent [1]. While most *E. coli* strains are harmless commensals, certain pathogenic strains can cause severe foodborne illnesses characterized by diarrhea, abdominal pain, and vomiting, and in severe cases may lead to kidney failure and other life-threatening complications [1,2]. Wastewater treatment plants (WWTPs) play a critical role in reducing the environmental dissemination of *E. coli* and other pathogens by removing microbial contaminants from sewage before treated effluent is discharged into natural water bodies [2]. However, conventional treatment processes may not eliminate pathogenic or antimicrobial-resistant bacteria, allowing them to persist and spread in the environment [1,2].

Among antimicrobial-resistant pathogens, extended-spectrum β-lactamase (ESBL)-producing *E. coli* has emerged as one of the most clinically significant due to its ability to hydrolyze a broad range of β-lactam antibiotics, including third-generation cephalosporin [1,3]. The global increase in ESBL-producing *E. coli* has been linked to treatment failure, prolonged hospital stays, increased healthcare costs, and higher mortality rates, making it a major public health concern [3,4,5]. The persistence of these resistant strains in wastewater and agricultural environments further highlights their role as important environmental reservoirs for the dissemination of antimicrobial resistance and virulence determinants [6].

Wastewater treatment plants (WWTPs) serve as critical environmental reservoirs and dissemination hubs for antibiotic-resistant bacteria and their associated resistance genes [5,7]. These facilities receive a continuous influx of domestic, industrial, and agricultural runoff; they naturally aggregate high concentrations of residual pharmaceuticals and diverse microbial communities [5,7,8]. As a result, resistant strains can survive in this toxic mixing bowl while vulnerable strains die [9]. This phenomenon is particularly evident in WWTPs, where *E. coli* and other bacteria are exposed to a variety of antibiotics and chemicals [8,9]. Over time, these conditions can lead to the emergence of antibiotic-resistant E. coli strains, posing a significant threat to public health [9,10]. These resistant bacteria can spread into the environment when treated wastewater is released into rivers and oceans [9,10,11,12]. They can also contaminate soil and crops when sludge from treatment plants is used as fertilizer [9,13]. This creates a pathway for antibiotic-resistant strains to enter the food chain, increasing the risk of infections in humans and animals [14,15]. Furthermore, the close physical proximity of microbes within treatment tanks facilitates horizontal gene transfer, meaning harmless bacteria can rapidly acquire resistance traits from pathogens [8,13].

Beyond mere antibiotic resistance, the true pathogenic potential of these environmental strains is heavily driven by virulence-associated genes and their ability to form resilient biofilms [9,13]. Virulence factors including adhesins, siderophores, toxins, invasins, and serum resistance proteins enhance bacteria colonization, host invasion, and survival within the wastewater treatment conditions [8,13,16]. These traits allow the bacteria to adapt and persist across diverse ecological niches far beyond treatment facilities [8,13,16]. Notably, comparative genomic analyses reveal that wastewater-derived strains often harbor virulence determinants comparable to clinical isolates, underscoring their potential public health risk [16,17,18].

Despite increasing reports of ESBL-producing *E. coli* in environmental settings, limited information exists on the combined role of virulence gene profiles and biofilm-forming capacity in their persistence and dissemination [19]. Understanding these characteristics is essential for assessing environmental health risks, improving surveillance strategies, and developing effective interventions aimed at mitigating the spread of antimicrobial-resistant pathogens [13,20]. Therefore, investigating the virulence determinants and biofilm-forming capacity of ESBL-producing *E. coli* from environmental sources can provide important insights into their persistence and potential public health impact [16,18,19,21].

This study aimed to characterize the virulence profiles and biofilm-forming abilities of ESBL-producing *E. coli* isolated from wastewater and agricultural environments in Fayetteville, North Carolina. Specifically, we evaluated how virulence factors and biofilm formation contribute to the environmental persistence and dissemination of antimicrobial-resistant *E. coli* across human, animal, and environmental interfaces within a One Health framework.

## 2. Materials and Methods

### 2.1. Area of Study

A total of 452 samples were obtained from environmental sources in Fayetteville, North Carolina, during a 12-month sampling period from May 2025 through May 2026. The sampling framework encompassed two municipal wastewater treatment facilities and two agricultural environments. At the treatment plants, 88 and 80 samples were collected from WWTP A and WWTP B, respectively. Agricultural sampling included 129 specimens from a beef cattle operation and 155 from a mixed-species backyard poultry operation housing chickens and ducks. Across all sampling locations, the sample set consisted of 168 wastewater samples, 128 animal fecal samples, 126 farm-water samples, and 30 soil samples. The disproportionately higher number of fecal samples reflects the limited availability of suitable water and soil sampling points at the farm sites, whereas fecal material was consistently present and readily obtainable.

### 2.2. Sample Collection

Samples were collected biweekly from two wastewater treatment plants (WWTPs), a cattle farm, and a poultry farm. At each WWTP, influent, effluent, sludge, and environmental sponge swab samples from high-contact surfaces were collected to assess microbial contamination across treatment stages. Wastewater samples were obtained using grab and composite sampling methods and collected in sterile 1 L polypropylene bottles from accessible effluent, influent, and sludge drainage points. Fecal samples were obtained using sterile scoops and transferred into sterile containers. Farm-water samples were collected using sterile bottles, and soil samples were taken from the top 2–5 cm using sterile spatulas and placed into sterile bags. At the cattle farm, fecal, water (lake and drinking trough), and soil samples were collected, while fecal and drinking-trough water samples were obtained from the poultry farm. All specimens were immediately placed on ice and transported to the laboratory within two hours.

### 2.3. Isolation and Identification of E. coli

Environmental samples were cultured on MacConkey agar and MacConkey agar (Becton, Dickinson and company, Sparks, MD, USA) supplemented with 1 µg/mL cefotaxime to isolate presumptive *E. coli* and ESBL-producing *E. coli*. Plates were incubated aerobically at 37 °C for 24 h. For effluent samples, all colonies were enumerated, whereas for influent, sludge, fecal, and soil samples, only plates containing 30–300 colony-forming units (CFUs) were included. Representative colonies from each sample were selected for further characterization. Presumptive *E. coli* isolates were streaked onto Eosin Methylene Blue (EMB) agar (Oxoid, Basingstoke, UK) and incubated at 37 °C for 24 h. Colonies exhibiting the characteristic metallic green sheen were subjected to biochemical confirmation using indole, sulfide-indole-motility (SIM), lysine decarboxylase, Simmons citrate, and Triple Sugar Iron (TSI) tests following standard microbiological procedures. Isolates demonstrating biochemical profiles consistent with *E. coli* were considered confirmed and subsequently subculture on Tryptic Soy Agar (Oxoid, Basingstoke, UK) to obtain pure cultures for downstream phenotypic and molecular analyses. All isolates in this study were newly collected and processed independently.

### 2.4. Screening for ESBL Production

Phenotypic confirmation of ESBL production was performed using the Clinical and Laboratory Standards Institute (CLSI) combined disk diffusion method [22]. Ceftazidime (30 µg), ceftazidime–clavulanic acid (30/10 µg), cefotaxime (30 µg), and cefotaxime–clavulanic acid (30/10 µg) disks (Becton, Dickinson and company, Sparks, MD, USA) were placed on the agar surface at CLSI-recommended spacing, and plates were incubated at 37 °C for 16–18 h. Following incubation, inhibition zone diameters were measured and interpreted according to CLSI criteria [22]. Isolates exhibiting an increase of ≥5 mm in zone diameter for either ceftazidime or cefotaxime in the presence of clavulanic acid compared with the corresponding antimicrobial alone were classified as ESBL producers. *Klebsiella pneumoniae* ATCC 700603 and *E. coli* ATCC 25922 served as ESBL-positive and ESBL-negative quality control strains, respectively.

### 2.5. Assessment of Virulence Signatures

#### 2.5.1. Multiplex PCR Amplification and Gel Electrophoresis

Multiplex polymerase chain reaction (PCR) assays, adapted from previous studies, were performed to detect virulence-associated genes in environmental *E. coli* isolates [23,24]. There were four groups of multiplex PCR, A, B, C, and D (Table 1). The target genes for multiplex PCR assay A included *iucD*, and *chuA*, (biosynthesis and haem receptor) which are associated with siderophore production, iron acquisition, and virulence potential. Multiplex PCR assay B targeted *ompA* and *traT* which are involved in pathogenicity and bacterial survival. Multiplex PCR assay C targeted *eaeA* and *stx1* which are involved in Intimin production and Shiga toxin 1 detection. PCR assay D targeted *fimH* and *Bfp* which are present on the surface of many *E. coli* cells and bind to the host intestinal lining. The assay D was validated in-house using gradient PCR. Primers used in this study were selected from previously published and validated multiplex PCR assays, as summarized in Table 1.

Before using the primer’s working stocks, they were diluted to a final concentration of 10 µM before being used. The master mixes were prepared on ice according to the virulence multiplex PCR protocol. Each 25 µL reaction contained molecular-grade water, DreamTaq PCR Master Mix (2×) (Thermo Fisher Scientific, Waltham, MA, USA), the appropriate forward and reverse primers for each target virulence gene, and 1 µL of DNA template. Positive and negative controls were included in every PCR run to verify amplification performance and monitor for potential contamination. Four PCR assays (PCR A–D) were performed using target-specific thermal cycling conditions. For PCR A, amplification consisted of an initial denaturation at 95 °C for 3 min, followed by 30 cycles of denaturation at 94 °C for 30 s, annealing at 59 °C for 60 s, and extension at 72 °C for 60 s. A final extension was performed at 72 °C for 5 min. For PCR B, the initial denaturation was performed at 94 °C for 5 min, followed by 30 cycles of denaturation at 94 °C for 1 min, annealing at 58 °C for 30 s, and extension at 68 °C for 3 min. A final extension was then performed at 72 °C for 10 min. For PCR C, amplification began with an initial denaturation at 95 °C for 5 min, followed by 35 cycles of denaturation at 95 °C for 30 s, annealing at 55 °C for 30 s, and extension at 72 °C for 3 min. The reaction concluded with a final extension at 72 °C for 7 min. For PCR D, an initial denaturation was performed at 95 °C for 5 min, followed by 35 cycles of denaturation at 94 °C for 30 secs, annealing at 55 °C for 30 secs, and extension at 72 °C for 1 min. A final extension was performed at 72 °C for 7 min. All PCR assays were conducted using a SimpliAmp™ Thermal Cycler (Thermo Fisher Scientific, Waltham, MA, USA).

PCR products from PCR A and PCR B were resolved by electrophoresis on 1.5% agarose gels, whereas products from PCR C and PCR D were separated on 2% agarose gels. Gels were prepared with SYBR Safe DNA Gel Stain (Thermo Fisher Scientific, Carlsbad, CA, USA), using 5 µL of SYBR Safe stain per 50 mL of molten agarose in accordance with the laboratory protocol. Following amplification, PCR products and an Invitrogen TrackIt™ 1 Kb Plus DNA Ladder (Thermo Fisher Scientific, Vilnius, Lithuania) were loaded onto the corresponding agarose gels and electrophoresed at 100 V for approximately 2 h.

Following electrophoresis, DNA bands were visualized using an Invitrogen iBright™ FL1500 Imaging System (Thermo Fisher Scientific, Waltham, MA, USA) under nucleic acid imaging settings. Amplicon sizes were estimated by comparison with the molecular-weight ladder and assessed against the expected fragment sizes for the respective virulence targets. Gel images were captured and manually interpreted. Isolates displaying a distinct amplification band at the expected molecular weight were considered positive for the corresponding virulence gene.

#### 2.5.2. DNA Extraction, Library Preparation, and Whole-Genome Sequencing

A subset of phenotypically confirmed ESBL-producing *E. coli* isolates (*n* = 53) was selected for whole-genome sequencing (WGS) based on source representation and funding availability. Genomic DNA was extracted using the DNeasy PowerLyzer Microbial Kit (Qiagen, Germantown, MD, USA) following the manufacturer’s protocol. Briefly, bacterial biomass was suspended in Power Bead and SL solutions and mechanically lysed using a Bead Mill 24 homogenizer (Omni, Kennesaw, GA, USA). Following centrifugation, DNA was purified through sequential treatment with IRS and SB solutions, captured on spin columns, washed with CB solution, and eluted in 50 μL of EB buffer. DNA quantity and purity were determined using a NanoDrop spectrophotometer (Thermo Fisher Scientific, Waltham, MA, USA).

Sequencing libraries with an average insert size of approximately 350 bp were prepared using the Illumina DNA Prep Kit and DNA/RNA UD Indexes (Illumina, San Diego, CA, USA). Pooled libraries were sequenced on an Illumina NextSeq 2000 platform using the NextSeq 1000/2000 P1 Reagent Kit (600 cycles), generating paired-end reads of 300 bp.

Raw sequence reads were processed through the MicroRunQC pipeline on the GalaxyTrakr platform for quality assessment and de novo genome assembly [25,26]. Genome assembly statistics, including genome size, contig count, N50, sequence type (ST), median insert size, and average read length, were generated for each isolate. All genomes satisfied established quality criteria for read coverage, assembly quality, and contamination screening. Species identification was verified using genome-based approaches, including average nucleotide identity (ANI) analysis and phylogenomic comparison with reference *E. coli* genomes. Virulence-associated genes were identified using VirulenceFinder database while sequence types were determined through multilocus sequence typing (MLST) analyses [27,28].

### 2.6. Biofilm-Forming Capacity Assay

Biofilm formation was examined by using the crystal violet microtiter plate assay. Pure isolates were streaked onto Tryptone Soy Agar and incubated at 37 °C for 18–24 h. A single colony was suspended in sterile diluent and adjusted to a 0.5 McFarland standard. Positive and negative controls were included throughout the assay. Biofilm medium was prepared using 5× M9 medium supplemented with casamino acids, MgSO_4_, CaCl_2_, thiamine, and glucose. Ninety microliters of medium and 10 µL of standardized inoculum were added to each well of sterile 96-well polystyrene microplates. Each isolate was tested in triplicate. Plates were incubated statically at 37 °C for 18–24 h. After incubation, planktonic cells were removed, and wells were gently blotted. Stained biofilms were solubilized with 100% ethanol, and optical density at 570 nm (OD_570_) was measured using a Tecan Sunrise microplate reader (Tecan, Mannedorf, Switzerland). Values outside the linear detection range were diluted and reanalyzed. Biofilm production was categorized relative to the negative control as non-biofilm-forming, weak, moderate, or strong formers.

### 2.7. Data Analysis

The prevalence and distribution of ESBL-producing *E. coli* were determined by sampling site and sample type using descriptive statistics. PCR results were recorded as presence or absence of targeted virulence genes. Associations between sampling source and virulence genes were assessed using Fisher’s exact test. Associations between biofilm category and virulence gene presence were evaluated using a chi-square test of independence. Benjamini–Hochberg FDR correction was applied to WGS-derived source–gene associations. PCR-based associations (8-gene panel) were evaluated using raw *p*-values without FDR adjustment; FDR < 0.05 was considered significant. Virulence gene richness was compared between sources using box-and-whisker plots with jittered points to display individual observations. Principal component analysis (PCA) was performed on WGS-derived virulence gene profiles using binary presence/absence data for all detected virulence genes. Prior to analysis, the binary matrix was centered but not scaled, as all variables were measured on the same scale. PCA was conducted in R v4.5.2 using the *prcomp()* function from the base *stats* package. The first two principal components (PC1 and PC2) were visualized to assess clustering patterns among isolates from cattle farms, poultry farms, WWTP-A, and WWTP-B.

Raw sequencing data for all isolates were submitted to the National Center for Biotechnology Information (NCBI) under BioProject PRJNA293225, with unique BioSample and SRA accession numbers assigned to each isolate. Biofilm formation was assessed using optical density values from the microtiter plate assay, with isolates classified as weak, moderate, or strong producers based on established cutoffs. Differences in biofilm-forming capacity between municipal wastewater and farm isolates were evaluated using appropriate statistical tests, with *p* < 0.05 considered significant. All analyses were performed using R v4.5.2 and Microsoft Excel.

## 3. Results

### 3.1. Distribution of Virulence Genes in ESBL E. coli

**Figure 1:**
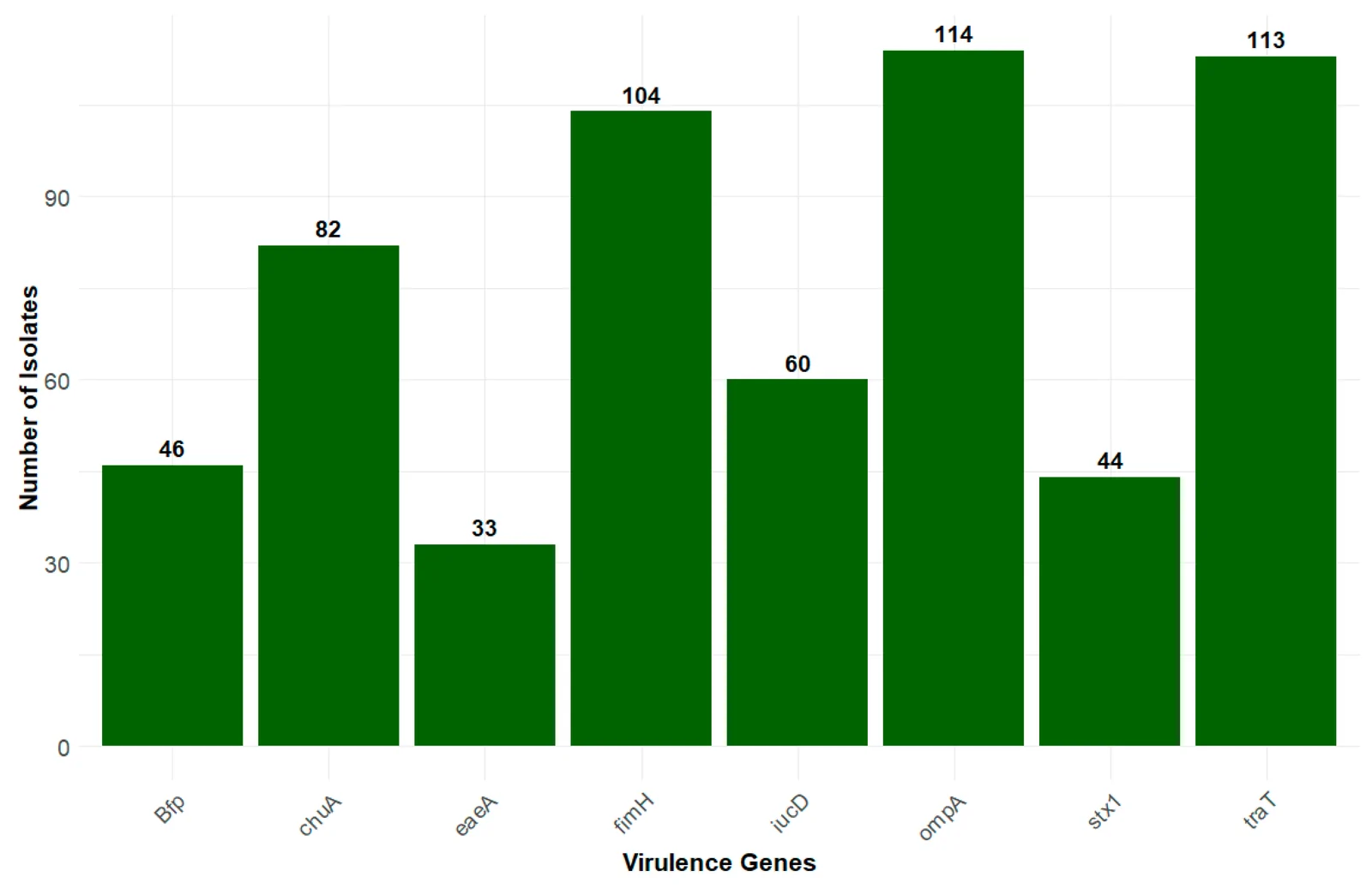
**Distribution of virulence-associated genes among ESBL-producing *E. coli* isolates.** Bar chart shows the frequency of PCR-detected virulence genes among ESBL-producing *E. coli* isolates. Bars represent the total number of isolates carrying each virulence gene, and the values above the bars indicate the corresponding gene counts. The predominance of adhesion- and colonization-associated genes (*ompA*, *traT*, and *fimH*) highlights the virulence potential of the ESBL-producing *E. coli* isolates. In contrast, *eaeA* was the least frequently detected virulence gene.

Of the 452 samples analyzed, 120 (26.5%) yielded ESBL-producing *E. coli*. The distribution of virulence-associated genes among ESBL-producing *E. coli* isolates is presented in Figure 1. Overall, *ompA* (114/120; 95%) and *traT* (113/120; 94.2%) were the most frequently detected genes, followed by *fimH* (104/120; 86.7%) and *chuA* (82/120; 68.3%). PCR analysis revealed that poultry farm isolates harbored the highest prevalence of virulence genes, particularly *traT* (64/66), *ompA* (61/66), *fimH* (54/66), and *chuA* (39/66).

### 3.2. Distribution of Virulence Genes by Location

**Figure 2:**
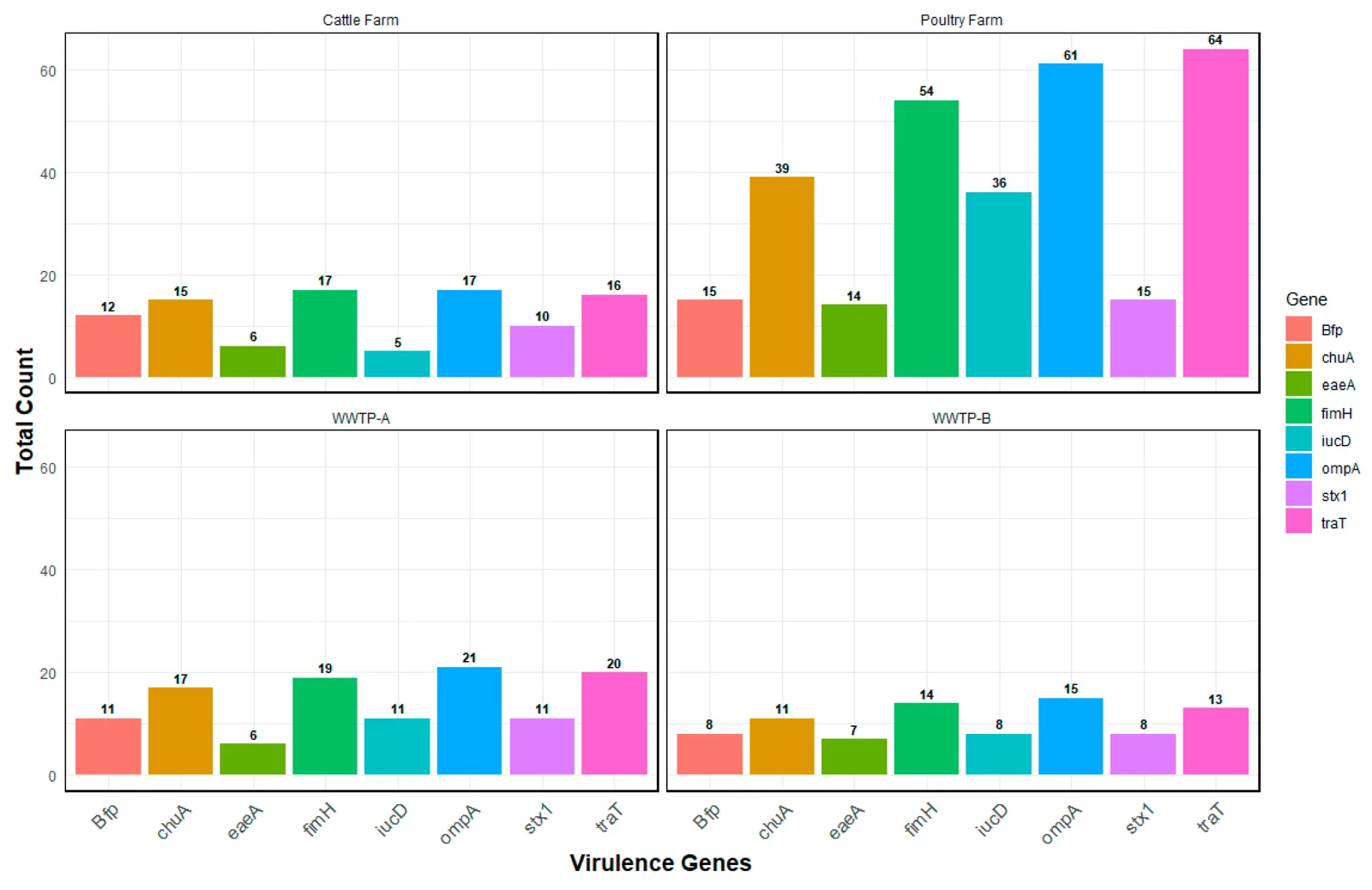
**Distribution of virulence-associated genes among ESBL *E. coli* isolates across sampling locations.** Bar plots showing the distribution of PCR-detected virulence genes among isolates recovered from four sampling locations: a cattle farm, a poultry farm, WWTP-A, and WWTP-B. Each panel represents a sampling location, and bars indicate the total number of isolates carrying each virulence gene. Values displayed above the bars correspond to the gene counts. The poultry farm exhibited the highest overall frequency of virulence genes, particularly *traT*, *ompA*, *fimH*, and *chuA*, followed by WWTP-A. In contrast, isolates from WWTP-B and the cattle farm carried comparatively fewer virulence-associated genes. This distribution highlights location-specific differences in the virulence gene repertoire among the isolates.

The distribution of virulence-associated genes varied across sampling locations (Figure 2). Overall, isolates from the poultry farm exhibited the highest frequency of virulence genes, followed by WWTP-A, cattle farm, and WWTP-B. Among poultry farm isolates (*n* = 66), *traT* (64/66; 96.9%), *ompA* (61/66; 92.4%), *fimH* (54/66; 81.8%), and *chuA* (39/66; 59.1%) were the most prevalent.

Similarly, among WWTP-A isolates (*n* = 22), *ompA* (21/22; 95.5%), *traT* (20/22; 90.9%), and *fimH* (19/22; 86.4%) were highly prevalent, whereas isolates from the cattle farm and WWTP-B exhibited comparatively lower frequencies. Across all locations, *eaeA* was the least frequently detected virulence gene.

Comparison of WGS-derived virulence gene prevalence among isolates from different sources revealed significant differences for several genes after false discovery rate (FDR) correction (FDR < 0.05). The genes *esp* (FDR = 3.47 × 10^−6^) and *fimH* (FDR = 2.36 × 10^−5^) showed the strongest associations with isolate source. Significant differences were also observed for *yagV*/*ecpE* (FDR = 3.34 × 10^−4^), *iroB* (FDR = 4.21 × 10^−4^), *fes* (FDR = 4.62 × 10^−4^), *ykgK*/*ecpR* (FDR = 2.26 × 10^−3^), *gspC* (FDR = 2.60 × 10^−3^), *fepA* (FDR = 3.04 × 10^−3^), *ent* (FDR = 1.66 × 10^−2^), and *fdeC* (FDR = 1.93 × 10^−2^).

In contrast, no significant differences were detected for *hlyA* (FDR = 0.147), *csgB* (FDR = 0.174), *gtrA* (FDR = 0.174), *stx2A*/*B* (FDR = 0.174), *ompA* (FDR = 0.335), *aslA* (FDR = 0.360), *chuA* (FDR = 0.360), *eae* (FDR = 0.764), or *shuA* (FDR = 0.896). Overall, source-associated variation in virulence gene content was primarily driven by differences in adhesin- and iron acquisition-related genes, whereas major virulence determinants such as *eae*, *stx2A*/*B*, and *hlyA* did not differ significantly among sources (Supplementary Table S1).

### 3.3. Distribution of Virulence Genes Across Environmental Sample Sources

**Figure 3:**
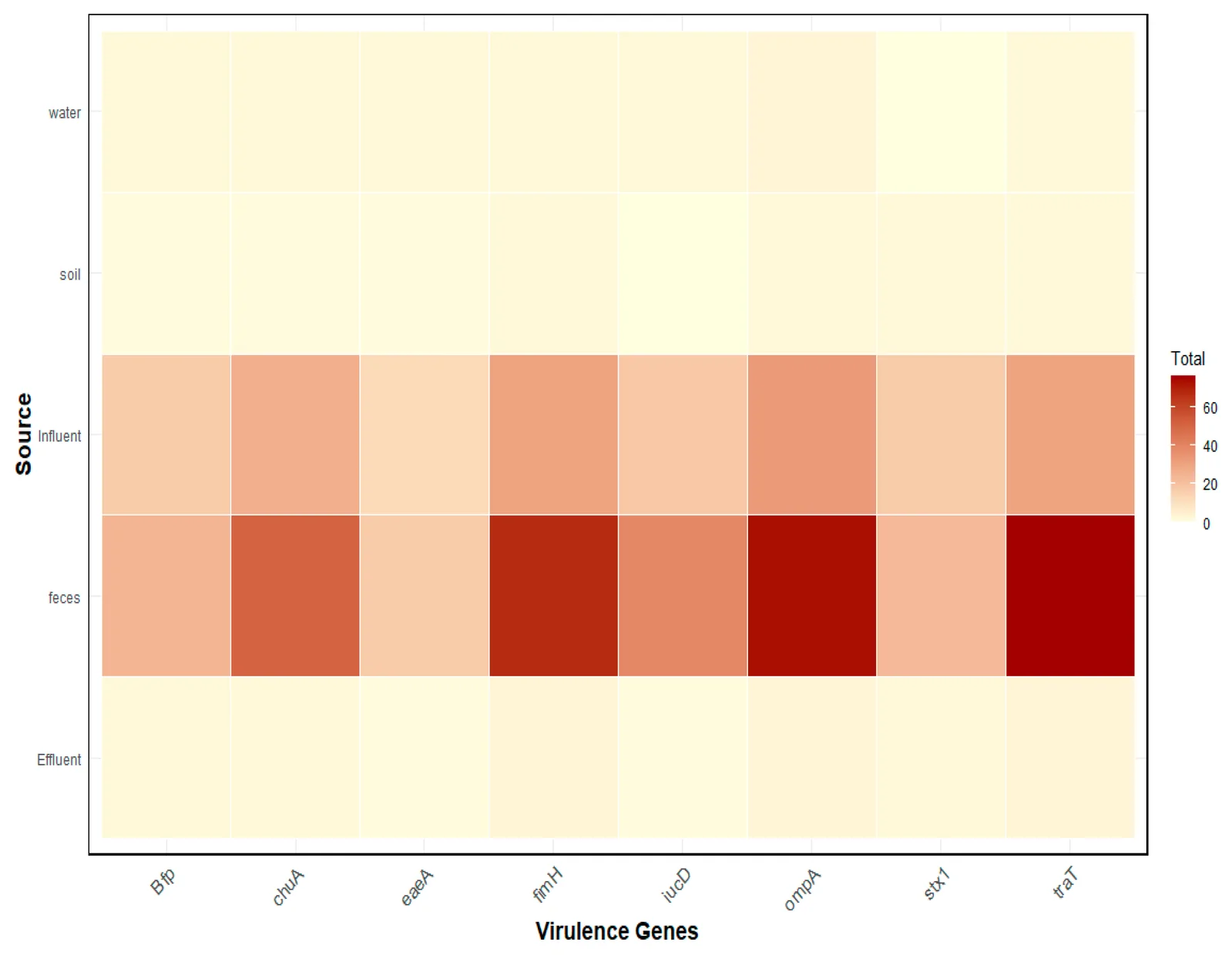
**Heatmap showing the distribution of PCR-derived virulence genes of ESBL *E. coli* across environmental sample sources.** This chart illustrates the distribution of virulence-associated genes among isolates recovered from different environmental sample sources, including influent, effluent, feces, soil, and water. Color intensity represents the total number of isolates carrying each virulence gene within a given sample source, with darker shades indicating higher frequencies. The heatmap demonstrates enrichment of virulence genes in fecal and influent isolates, whereas effluent, soil, and water isolates exhibited comparatively lower frequencies of the virulence determinants analyzed.

The distribution of virulence genes varied markedly across sample sources (Figure 3). Overall, fecal isolates exhibited the highest prevalence of virulence determinants, followed by influent wastewater isolates, whereas isolates recovered from effluent, soil, and water samples carried comparatively fewer virulence genes. Among the genes detected, *traT*, *ompA*, *fimH*, and *chuA* were the most frequently identified, with the greatest abundance observed in fecal isolates. Influent isolates also harbored a broad repertoire of virulence genes, although at lower frequencies than fecal isolates. In contrast, effluent, soil, and water isolates showed uniformly low frequencies across all virulence genes examined. These findings indicate that virulence-associated determinants are enriched in fecal and influent environments, suggesting that these sources may serve as important reservoirs of potentially pathogenic *E. coli*.

The prevalence of the *traT* and *stx1* genes varied significantly among sources (raw *p* = 0.0398 and raw *p* = 0.0224, respectively), suggesting differences in the distribution of serum resistance-associated and Shiga toxin virulence traits across sources.

### 3.4. Source-Dependent Differences in Virulence Gene Richness

**Figure 4:**
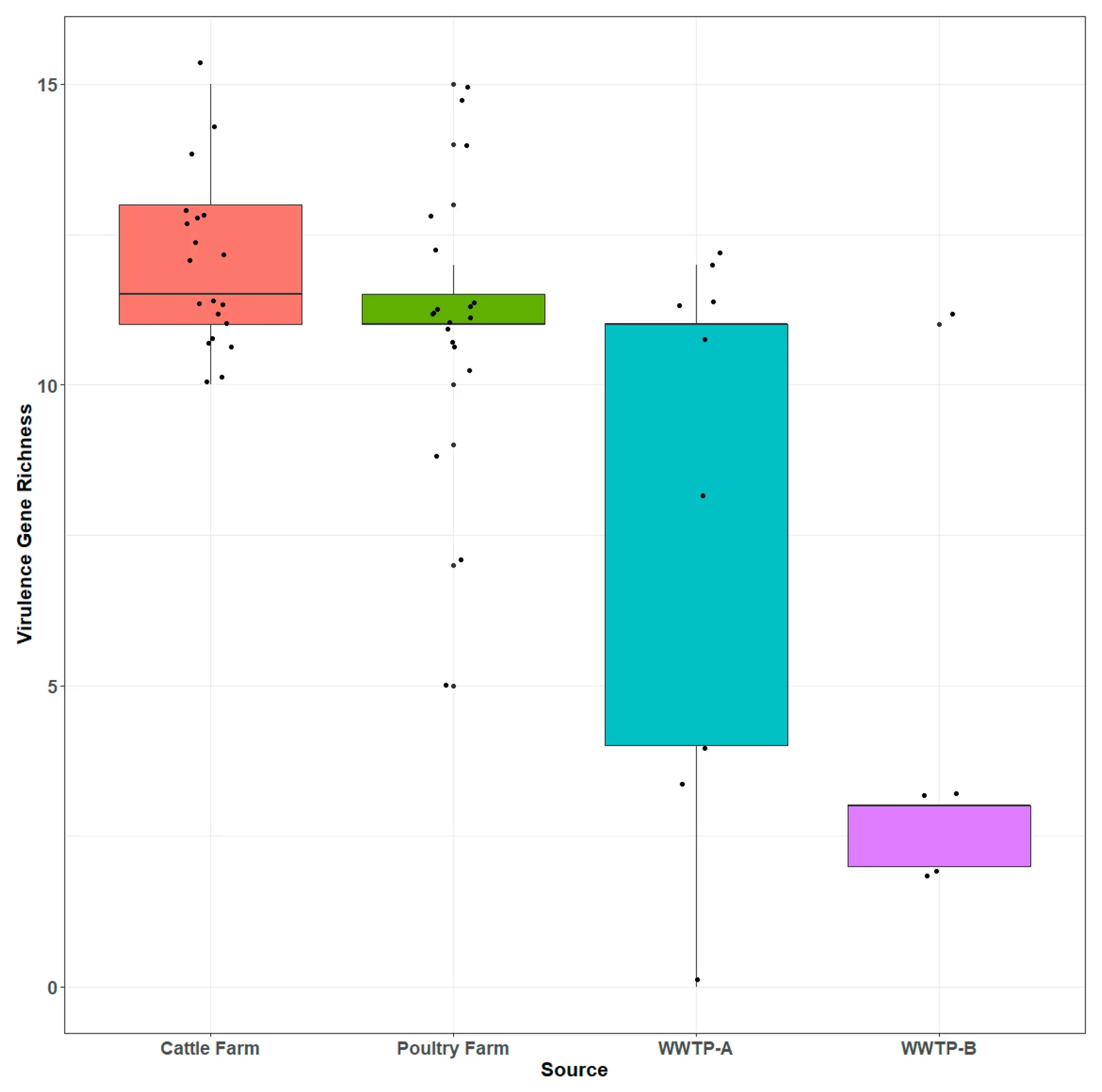
**Distribution of virulence gene richness among isolates from agricultural and municipal wastewater sources determined by whole-genome sequencing** (**WGS**)**.** Boxplots show the number of virulence-associated genes detected per isolate across four sources: cattle farms (*n* = 20), poultry farms (*n* = 19), WWTP-A (*n* = 9), and WWTP-B (*n* = 5). Boxes represent the interquartile range (IQR), the horizontal line within each box indicates the median, and whiskers extend to 1.5 × IQR. Individual points represent virulence gene richness values for each isolate. Black dots represent outliers, defined as observations lying beyond 1.5 × IQR from the upper or lower quartiles.

Virulence gene richness differed markedly among the four sample sources in the WGS subset (cattle farms, *n* = 20; poultry farms, *n* = 19; WWTP-A, *n* = 9; WWTP-B, *n* = 5) (Figure 4). Cattle farm isolates exhibited high richness, with median values around 11–12 genes and a broad distribution ranging from approximately 10 to 15 genes. Poultry farm isolates showed a similar median but greater variability, spanning roughly 5 to 15 genes. In contrast, isolates from WWTP-A showed substantial heterogeneity, with virulence gene richness ranging from 0 to about 12 genes and a median near 11 genes, indicating the presence of both highly enriched and low-richness isolates within this source. WWTP-B isolates had the lowest virulence gene richness overall, with scores largely clustered between 2 and 3 genes and only a few isolates exhibiting higher values around 11 genes. Agricultural sources (cattle and poultry farms) harbored isolates with consistently higher virulence gene richness compared with wastewater treatment plant sources, particularly WWTP-B.

### 3.5. Principal Component Analysis of Virulence Gene Profiles

**Figure 5:**
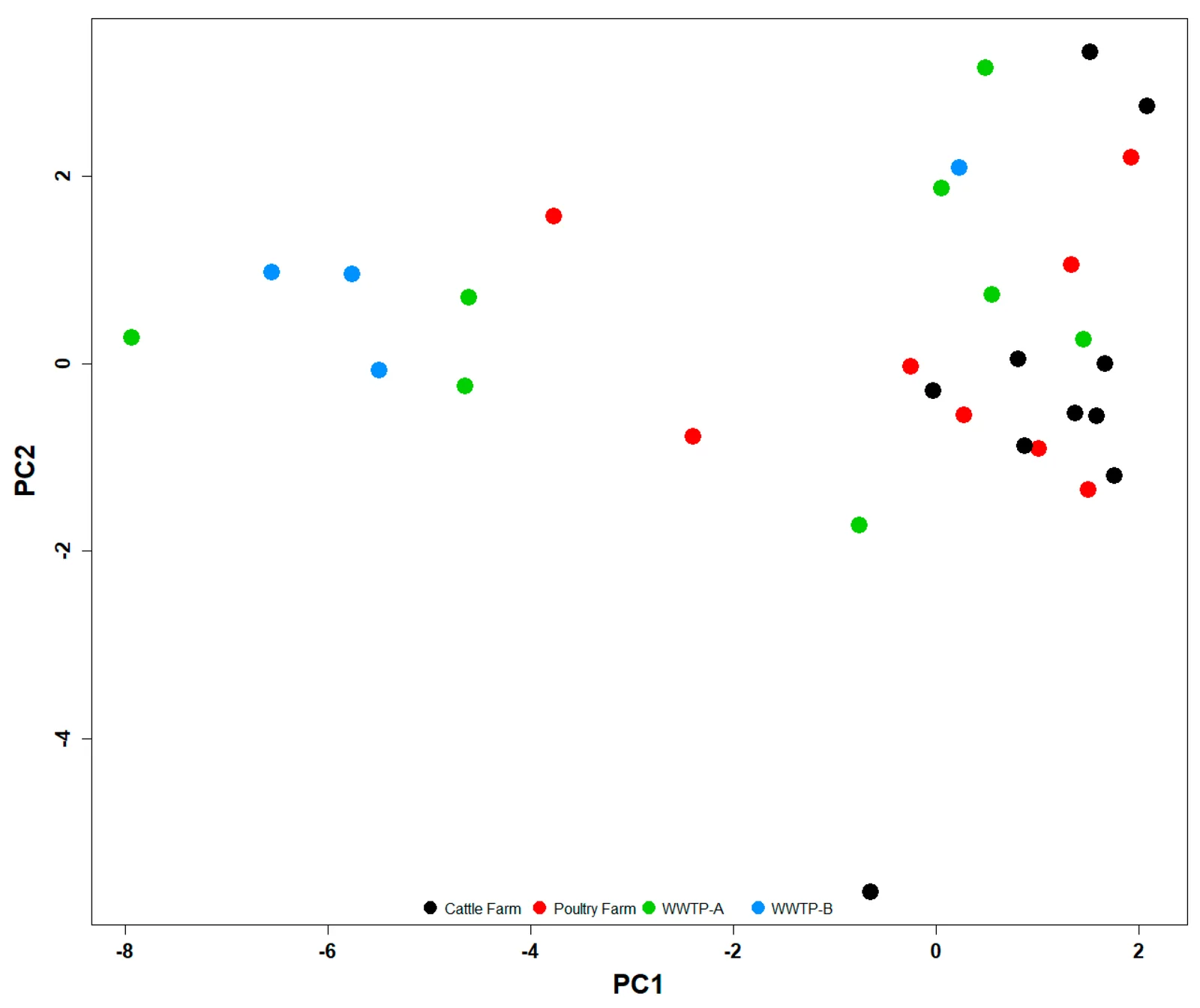
**Principal component analysis showing the distribution of WGS-derived virulence gene profiles among *E. coli* isolates from agricultural and wastewater environments.** Each point represents a single isolate and is colored according to its source (cattle farms, poultry farms, WWTP-A, and WWTP-B). The first two principal components (PC1 and PC2) summarize the variation in virulence gene composition among isolates. Partial clustering of isolates by source was observed, although substantial overlap occurred among groups, indicating similarities in virulence gene profiles across agricultural and wastewater environments. WWTP-A isolates displayed greater dispersion across the ordination space, while cattle farm isolates formed a relatively tighter cluster.

Principal component analysis (PCA) was performed to evaluate the similarity of WGS-associated virulence gene profiles among isolates from cattle farms, poultry farms, WWTP-A, and WWTP-B (Figure 5). The PCA revealed substantial overlap among isolates from the different sources, indicating that virulence gene composition was not completely source-specific. However, some clustering patterns were evident along the first principal component (PC1), with many cattle farm and poultry farm isolates grouped toward positive PC1 values, while several WWTP-A and WWTP-B isolates were distributed toward negative PC1 values. WWTP-A isolates exhibited the greatest dispersion across the PCA space, reflecting considerable heterogeneity in virulence gene composition within this source. In contrast, cattle farm isolates formed a relatively tighter cluster, suggesting more similar virulence gene profiles among isolates from this environment. The poultry farm and WWTP-B isolates showed intermediate levels of dispersion and partially overlapped with both farm- and wastewater-derived isolates. Several isolates appeared distant from the main clusters, indicating the presence of unique virulence gene profiles.

### 3.6. Distribution of Virulence Genes in ESBL-E. coli by Biofilm Categories

Across the isolate collection, biofilm formation varied substantially, with isolates classified as non-biofilm, weak, moderate, or strong producers (Table 2). Non-biofilm isolates constituted the largest group and exhibited the highest overall counts for most virulence genes, including *ompA*, *traT*, *iucD*, *chuA*, *stx1*, *fimH*, and *Bfp*. Weak biofilm producers also showed notable gene presence, particularly for *ompA*, *traT*, and *fimH.* Moderate biofilm producers displayed intermediate gene frequencies, whereas strong biofilm producers were few and carried comparatively lower counts of most virulence genes. Overall, virulence gene distribution differed across biofilm categories, with non-biofilm and weak biofilm isolates contributing most detected gene occurrences.

### 3.7. Biofilm-Forming Capacity Across Sampling Locations

**Figure 6:**
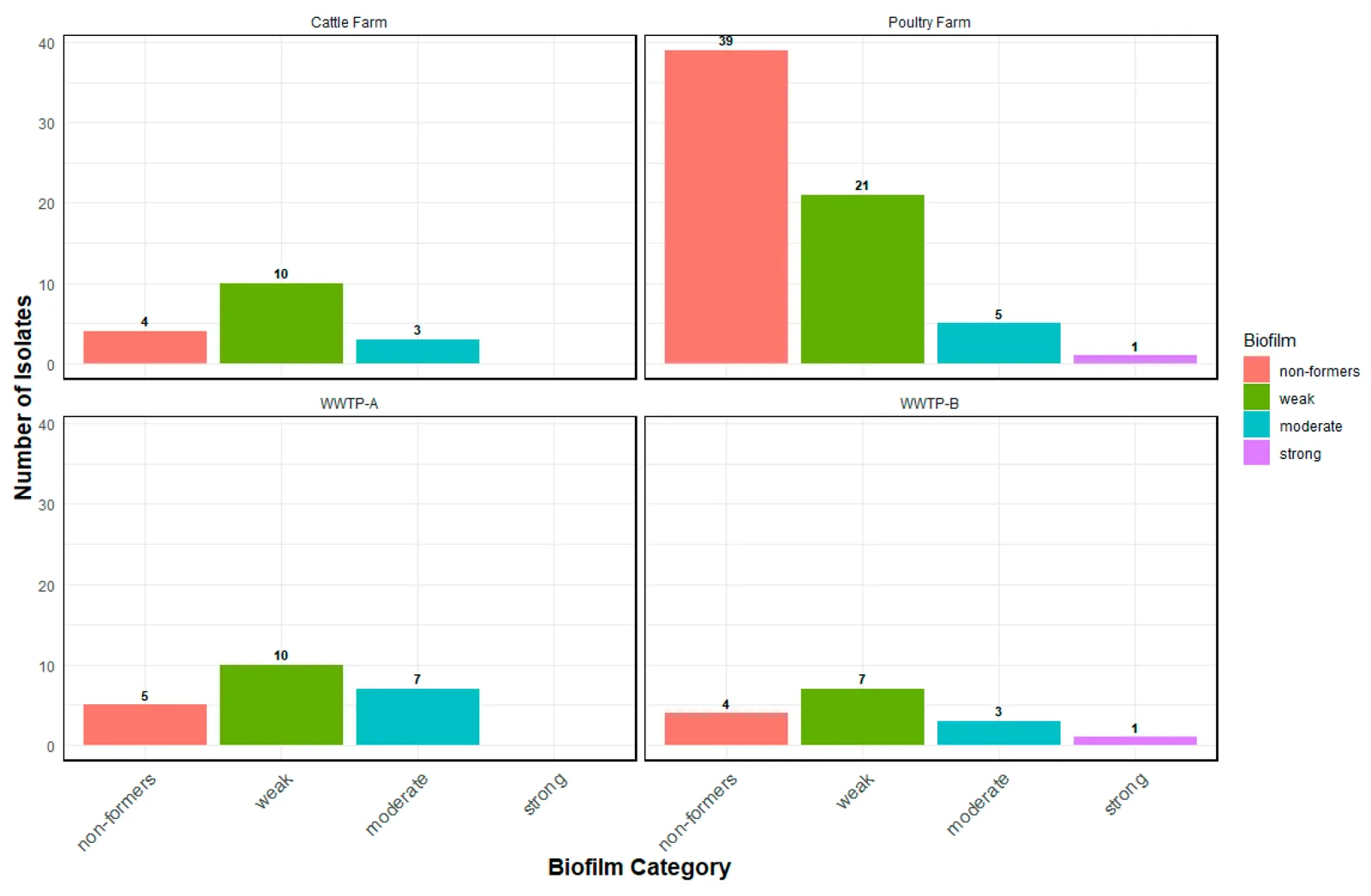
**Distribution of biofilm-forming phenotypes among ESBL-producing *E. coli* isolates across sampling locations.** Each panel represents a sampling location (cattle farm, poultry farm, WWTP-A, or WWTP-B), and bars indicate the number of isolates classified as non-biofilm formers, weak, moderate, or strong biofilm producers based on the crystal violet microtiter plate assay. Values above the bars represent the number of isolates in each biofilm category. Weak biofilm formation was the predominant phenotype across all locations, whereas strong biofilm-forming isolates were infrequently detected and were observed only in the poultry farm and WWTP-B.

The distribution of biofilm-forming phenotypes varied across sampling locations (Figure 6). Weak biofilm formation was the predominant phenotype among biofilm-producing isolates and was most frequently observed in the poultry farm (*n* = 21), followed by the cattle farm and WWTP-A (*n* = 10 each) and WWTP-B (*n* = 7). Moderate biofilm formation occurred less frequently, with the highest number of isolates detected in WWTP-A (*n* = 7), followed by the poultry farm (*n* = 5), cattle farm (*n* = 3), and WWTP-B (*n* = 3). Strong biofilm formation was rare and was detected only among isolates from the poultry farm (*n* = 1) and WWTP-B (*n* = 1). In contrast, non-biofilm producers constituted the largest phenotypic group overall, with the highest number observed in the poultry farm (*n* = 39), followed by WWTP-A (*n* = 5), and the cattle farm and WWTP-B (*n* = 4 each). Overall, strong biofilm formation was uncommon across all sampling locations.

*Bfp*, *eaeA*, *stx1*, *iucD*, and *traT* all showed statistically significant associations with biofilm category (*p* < 0.01), suggesting that strains capable of forming moderate to strong biofilms are more likely to possess genes involved in adhesion, toxin production, iron acquisition, and serum resistance. The strongest associations were observed for *Bfp* (*p* = 3.51 × 10^−7^), *eaeA* (*p* = 1.19 × 10^−4^), and *stx1* (*p* = 4.27 × 10^−5^), highlighting that biofilm-forming isolates frequently carry genes linked to intimate adherence and Shiga toxin production, traits often associated with enhanced pathogenic potential. In contrast, *chuA*, *fimH*, and *ompA* showed no significant association with biofilm strength (*p* > 0.05), indicating that these genes are more uniformly distributed across biofilm categories and may represent core traits not directly tied to biofilm formation.

### 3.8. Multilocus Sequence Typing (MLST)

MLST analysis identified considerable genetic heterogeneity among the sequenced ESBL-producing *E. coli* isolates (Supplementary Table S1). The predominant sequence type was ST602, detected exclusively in poultry isolates (*n* = 6), followed by ST2063 (*n* = 3). Among cattle isolates, several sequence types were represented by multiple isolates, including ST119, ST1704, ST2073, ST2387, ST278, and ST645 (*n* = 2 each). Wastewater-derived isolates were genetically diverse, with only ST636 occurring more than once (*n* = 2).

## 4. Discussion

The widespread detection of virulence-associated genes among ESBL-producing *E. coli* isolates underscores the capacity of environmental reservoirs to harbor strains with enhanced pathogenic potential. The predominance of *ompA*, *traT*, *fimH*, and *chuA* indicates that many isolates possess traits linked to adhesion, colonization, serum resistance, and persistence within host and environmental niches [3,29]. The high frequency of *fimH*, a type 1 fimbrial adhesin, aligns with its well-established role in epithelial attachment and biofilm formation, while *ompA* contributes to adhesion, invasion, and survival under environmental stress [18,30]. In contrast, the relatively low prevalence of *eaeA* and *stx1* suggests that classical diarrheagenic virulence determinants were uncommon among these environmental isolates, indicating that extraintestinal virulence mechanisms may predominate in ESBL-producing populations circulating in wastewater and agricultural settings [31,32].

Location-specific differences in virulence gene distribution further demonstrate how environmental conditions and selective pressures shape the virulence repertoire of ESBL-producing *E. coli* [33,34]. Poultry farm isolates exhibited the highest frequencies of virulence-associated genes, followed by isolates from WWTP-A, whereas cattle farm and WWTP-B isolates carried comparatively fewer virulence determinants [14,33,35]. Because the poultry site was a mixed-species backyard flock, not an intensive production facility, the observed virulence genes are best interpreted in the context of backyard poultry systems, where virulent and biofilm-forming *E. coli* have also been reported [36,37]. However, the factors driving their occurrence could not be determined because farm management and antimicrobial use data were not collected.

Wastewater treatment plants, in turn, receive inputs from human, agricultural, and industrial sources, creating dynamic environments that promote the accumulation, persistence, and exchange of genetic material among diverse bacterial populations [38,39]. The elevated frequency of virulence genes in WWTP-A supports the role of wastewater systems as important environmental reservoirs of potentially pathogenic *E. coli* [33,39].

The source-specific distribution of virulence genes reinforces these patterns. Fecal and influent wastewater isolates harbored the greatest diversity and abundance of virulence determinants, whereas isolates recovered from effluent, soil, and surface water carried substantially fewer virulence genes [13,39,40]. Feces represent the primary reservoir of intestinal *E. coli*, while influent wastewater concentrates bacteria originating from humans, animals, and surrounding environments [40]. These matrices therefore provide favorable conditions for the persistence and potential dissemination of virulent ESBL-producing strains [40,41]. Although virulence gene frequencies were lower in effluent samples, their continued detection following treatment indicates that potentially pathogenic *E. coli* may survive treatment processes and enter receiving environments, highlighting the need to optimize wastewater treatment strategies to reduce environmental dissemination [40,41,42]. Biofilm formation represents an additional factor contributing to the environmental persistence of antimicrobial-resistant bacteria [40,42,43].

The source-associated distribution of several adhesion-related genes, including *esp*, *fimH*, *fdeC*, *yagV*/*ecpE*, and *ykgK*/*ecpR*, in the present study suggests that ecological origin may influence traits involved in bacterial attachment, colonization, and persistence [18]. Rather than indicating differences in classical pathogenicity alone, these patterns may reflect adaptation to distinct host-associated or environmental niches, where efficient attachment and establishment can enhance bacterial survival [44,45]. Similar associations between adhesin profiles and ecological or host niches have been reported in *E. coli*, supporting the potential role of these factors in reservoir-specific adaptation [46,47].

The source-dependent distribution of iron acquisition genes, including *iroB*, *fepA*, *fes*, and *ent*, further suggests that iron availability may contribute to the ecological selection of *E. coli* populations [48]. Iron acquisition enhances bacterial fitness by supporting essential cellular processes under iron-limited conditions; source-specific iron uptake and siderophore genes may therefore promote persistence and competitiveness in distinct reservoirs [48,49].

Our results show that major virulence-associated genes, including *eae*, *stx2A*/*B*, and *hlyA*, did not differ significantly by sampling source. This finding suggests that the potential for pathogenicity may not be confined to a particular environmental or agricultural reservoir [31,47]. Instead, the observed source-associated differences may primarily reflect variation in colonization, persistence, and ecological fitness traits rather than classical virulence determinants [17,31,44]. These traits may enhance *E. coli* persistence, transmission, and horizontal gene transfer across reservoirs, supporting the inclusion of colonization- and fitness-associated genes alongside classical virulence markers in surveillance [18].

Our results showed that virulence gene richness varied significantly across source types. Isolates from cattle and poultry farms harbored the highest virulence gene richness, suggesting that agricultural environments may serve as important reservoirs of virulence-associated determinants and are consistent with reports from other studies [50,51]. In contrast, WWTP-B isolates exhibited the lowest richness, indicating a reduced burden of virulence genes. WWTP-A showed the greatest variability, with isolates ranging from no detectable virulence genes to richness levels comparable to those observed in farm-derived isolates. This heterogeneity may reflect diverse wastewater inputs and microbial populations [52,53,54]. The higher virulence gene richness observed in agricultural isolates compared with wastewater isolates highlights the influence of source environment on the distribution of virulence-associated genes and underscores the importance of monitoring these reservoirs for potential public health risks [55,56].

The substantial overlap in PCA profiles across cattle, poultry, and wastewater isolates in the present study suggests that virulence-associated determinants are broadly distributed across these interconnected reservoirs. Similar cross-source distributions of *E. coli* virulence factors have been reported in environmental, livestock, food, and human-associated isolates, supporting the potential for shared ecological niches and transmission pathways [18,31,44]. The tighter clustering of cattle isolates suggests greater similarity in their virulence profiles, whereas the broader dispersion of WWTP-A isolates may reflect greater heterogeneity arising from the diverse microbial inputs entering wastewater systems [57,58]. Wastewater can receive *E. coli* from multiple human and animal sources and may provide opportunities for persistence and genetic exchange [6,59]. The absence of clear separation among sources therefore supports the interconnectedness of agricultural and wastewater reservoirs and highlights their potential role in maintaining and disseminating virulence-associated determinants [6,58].

The predominance of weak biofilm producers across all sampling locations suggests that, despite the presence of multiple virulence-associated genes, most isolates exhibited limited biofilm-forming capacity. Although agricultural isolates possessed greater virulence gene richness than wastewater-derived isolates, this did not appear to translate into widespread strong biofilm formation. This finding highlights that virulence gene carriage alone may not predict biofilm phenotype, as biofilm development is influenced by the expression of specific adhesion- and biofilm-associated genes as well as environmental conditions [15,60,61]. Biofilms promote close bacterial interactions that facilitate horizontal gene transfer, including the exchange of antimicrobial resistance and virulence determinants [8,40,43]. Thus, the predominance of weak biofilm producers suggests that many environmental ESBL-producing *E. coli* isolates possess sufficient biofilm-forming capacity to survive within wastewater systems and agricultural environments, contributing to their persistence and dissemination [40,43,62,63,64].

The coexistence of antimicrobial resistance, multiple virulence determinants, and biofilm-forming ability underscores the public health significance of these environmental reservoirs [64]. These results highlight the need for integrated One Health surveillance approaches that incorporate genomic monitoring across environmental, animal, and human sectors to better characterize transmission pathways and inform interventions aimed at limiting the spread of antimicrobial-resistant and potentially pathogenic *E. coli* [63,64,65].

This study has several limitations. The investigation was conducted across a limited number of poultry and cattle farms, as well as wastewater treatment plants, which may restrict the broader applicability of the findings to other geographic locations or production settings. In addition, variations in sample availability and collection across sites, largely driven by logistical constraints and site accessibility, resulted in unequal representation of environmental matrices. Because sampling occurred over a single 12-month period, the study may not capture broader seasonal or long-term environmental variability. Budgetary limitations also restricted the number of isolates that could be subjected to sequencing, thereby limiting comprehensive genomic assessment of the entire isolate collection. As a result, the taxonomic identification of isolates that were not sequenced could not be verified at the genomic level and should be interpreted cautiously. Furthermore, isolates selected for sequencing were prioritized based on their source origin, which may introduce selection bias and affect the extent to which the sequenced subset represents the overall population of isolates recovered in this study.

## 5. Conclusions

This study demonstrates that wastewater treatment plants and livestock production environments serve as important reservoirs of ESBL-producing *E. coli* carrying diverse antimicrobial resistance determinants, virulence-associated genes, and biofilm-forming capabilities. The predominance of adhesion- and persistence-associated virulence genes, together with the widespread ability to form biofilms, highlights the capacity of these environmental isolates to survive, persist, and potentially contribute to the dissemination of antimicrobial resistance. The higher frequencies of virulence genes observed in poultry farms and wastewater isolates further emphasize the influence of environmental and agricultural settings on the maintenance of potentially pathogenic *E. coli*. Collectively, these findings reinforce the importance of adopting a One Health approach to monitor antimicrobial-resistant bacteria at the human–animal–environment interface.

## Figures and Tables

**Table 1:** **PCR protocols used for *E. coli* virulence gene detection.**

Virulence Genes	Forward—Primers DNA Sequence (5′ to 3′)	Reverse—Primers DNA Sequence (5′ to 3′)	Amplicon Sizes	References
PCR assay A				
*iucD*	TACCGGATTGTCATATGCAGACCG	AATATCTTCCTCCAGTCCGGAGAAG	714	[23]
*chuA*	GACGAACCAACGGTCAGGAT	TGCCGCCAGTACCAAAGACA	279	[23]
PCR assay B				
*ompA*	AGCTATCGCGATTGCAGTG	GGTGTTGCCAGTAACCGG	919	[23]
*traT*	GGTGTGGTGCGATGAGCACAG	CACGGTTCAGCCATCCCTGAG	290	[23]
PCR assay C				
*eaeA*	AGGCTTCGTCACAGTTG	CCATCGTCACCAGAGGA	570	[24]
*stx1*	TCTCAGTGGGCGTTCTTATG	TACCCCCTCAACTGCTAATA	388	[24]
PCR assay D				
*fimH*	TGCAGAACGGATAAGCCGTGG	GCAGTCACCTGCCCTCCGGTA	508	[24]
*Bfp*	AATGGTGCTTGCGCTTGCTGC	GCCGCTTTATCCAACCTGGTA	324	[23]

**Table 2:** **Virulence Gene Counts by Biofilm Category.**

Biofilm Category	*ompA*	*traT*	*iucD*	*chuA*	*eaeA*	*stx1*	*fimH*	*Bfp*
Moderate	17	16	5	12	6	12	16	11
Non-biofilm	50	52	34	36	5	8	45	6
Strong	1	1	0	1	2	1	2	1
Weak	46	44	21	33	20	23	41	28

## Data Availability

The data supporting the findings of this study are available within the manuscript and its Supplementary Materials and are publicly accessible through the National Center for Biotechnology Information (NCBI) under BioProject accession number PRJNA293225 (https://www.ncbi.nlm.nih.gov/bioproject/?term=PRJNA293225; accessed 15 July 2026).

## Supplementary Materials

The following supporting information can be downloaded at https://www.mdpi.com/article/10.3390/microorganisms14102239/s1, Supplementary Table S1. ESBL-EC strains used for Virulence genes and MLST analysis.
